# Supplementary material for: O-GlcNAcylation and Regulation of Galectin-3 in Extraembryonic Endoderm Differentiation
Source: Biomolecules. 2022 Apr 22;12(5):623. doi: 10.3390/biom12050623 (PMC9138951; doi:10.3390/biom12050623)
Supplement: Supplementary file 1 [file biomolecules-12-00623-s001.zip › biomolecules-1635795-supplementary.pdf]

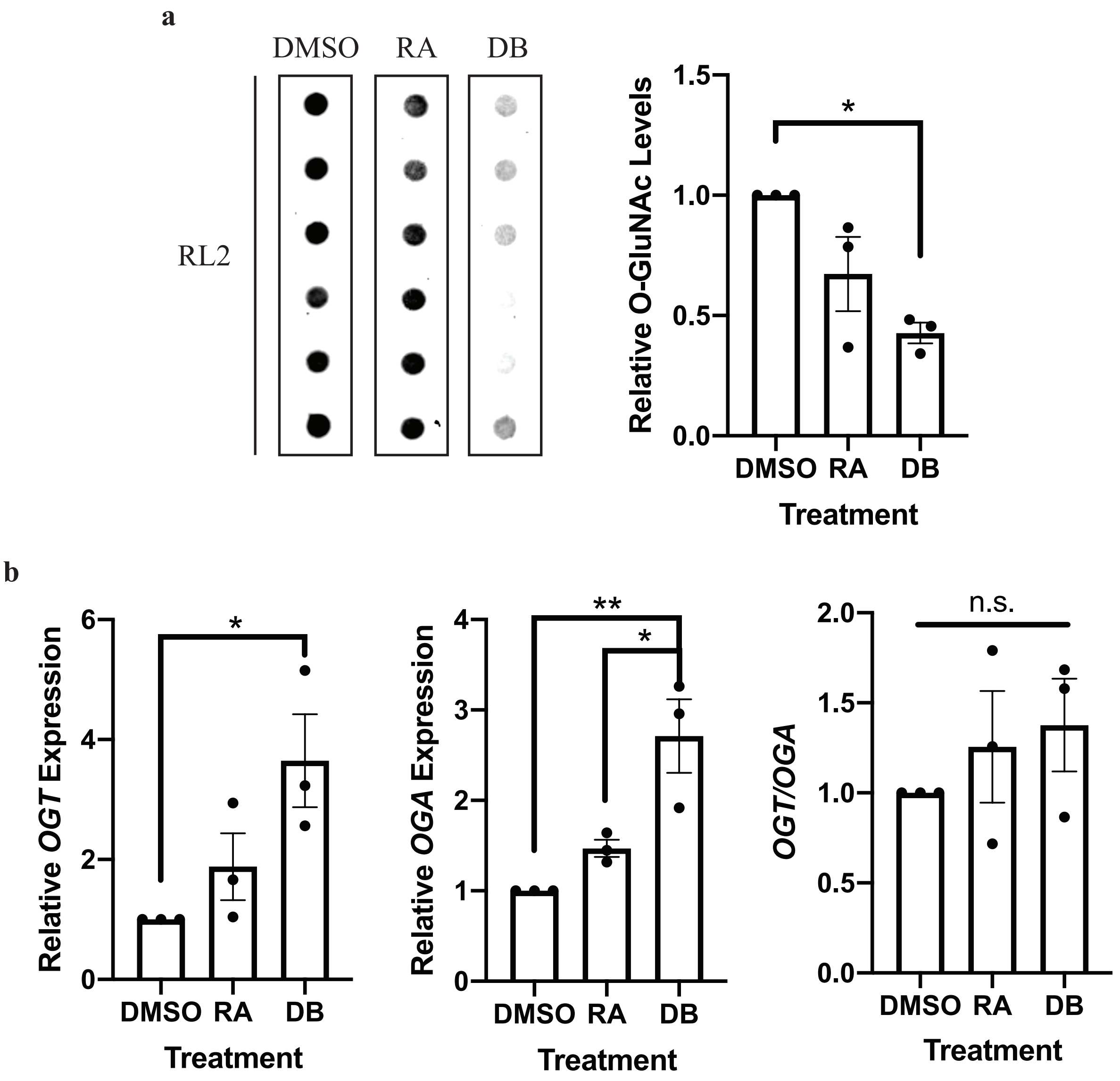

Supplementary Figure S1. Global *O*-GlcNAcylation decreases with F9 cell differentiation towards a XEN-like lineage: (**a**) Immunodot blot and densitometry of F9 cells treated with DMSO (control), 0.5μM RA, or 0.5μM RA + 1 mM db-cAMP; (**b**) Expression levels of *Ogt* and *Oga* transcripts in F9 cells treated as in (a) as detected by RT-qPCR and their ratio. Bars represent means values ± S.E.M, N=3. *P<0.05, **P<0.01.

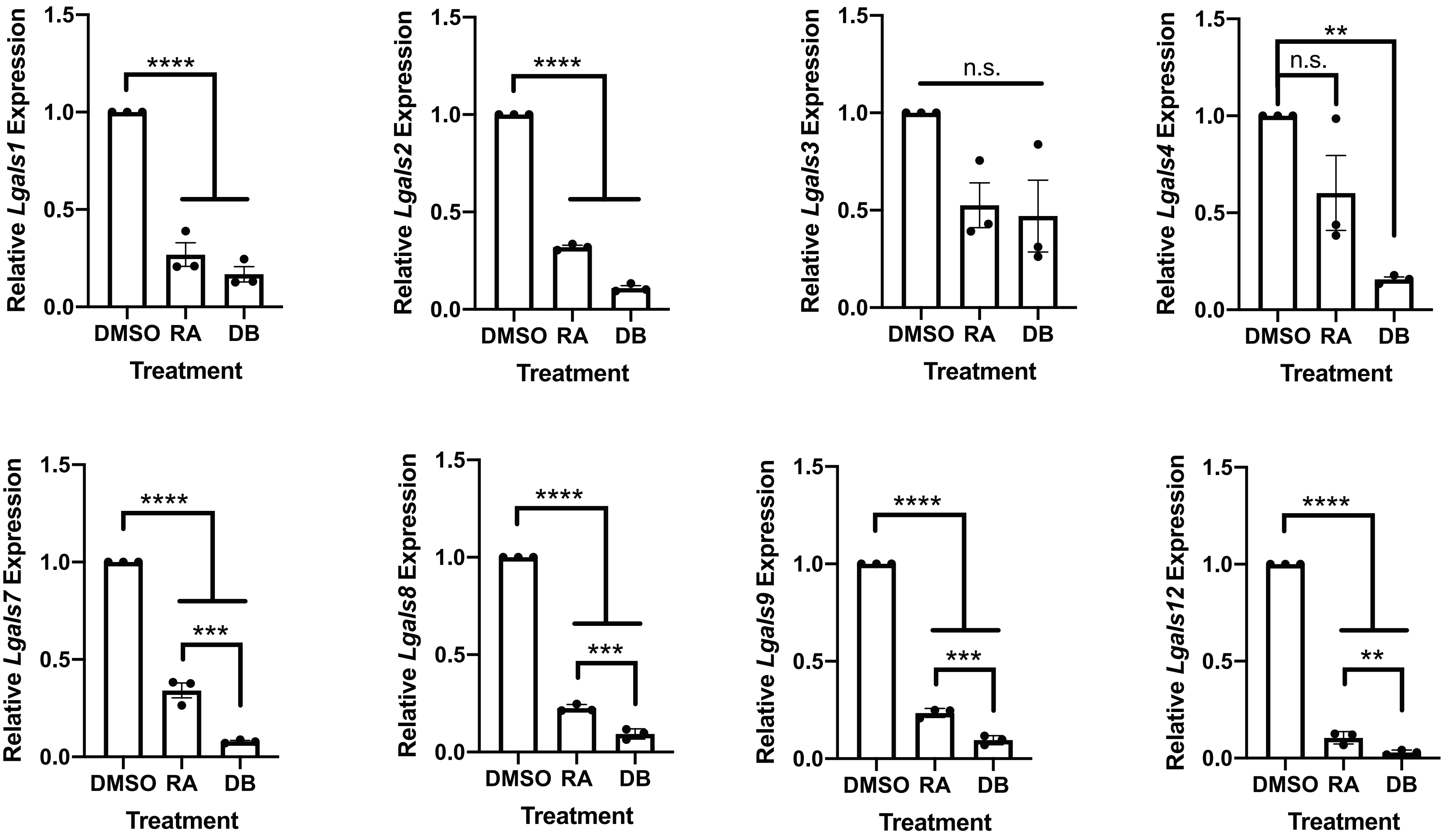

Supplementary Figure S2. Expression of many galectins are decreased in F9 cells differentiated towards a XEN-like lineage. Expression of *Lgals1*, *Lgals2*, *Lgals3*, *Lgals4*, *Lgals7*, *Lgals8*, *Lgals9* and *Lgals12* as detected by RT-qPCR in F9 cells treated with DMSO (control), 0.5 μM RA or 0.5 μM RA + db-cAMP. Bars represent mean values ± S.E.M, N=3. **P<0.01, ***P<0.001, ****P<0.0001.
